# Sedation and renal impairment in critically ill patients: a *post hoc *analysis of a randomized trial

**DOI:** 10.1186/cc10218

**Published:** 2011-05-04

**Authors:** Thomas Strøm, Rasmus R Johansen, Jens O Prahl, Palle Toft

**Affiliations:** 1 Department of Anesthesia and Intensive Care Medicine, Odense University Hospital, University of Southern Denmark, Sdr Boulevard 29, 5000 Odense C, Denmark

## Abstract

**Introduction:**

Not sedating critically ill patients reduces the time patients receive mechanical ventilation, decreases the time in the intensive care department and reduces the total hospital length of stay. We hypothesized that no sedation improves hemodynamic stability, decreases the need for vasoactive drugs, diminishes the need for extra fluids and lowers the risk of acute kidney injury.

**Methods:**

We performed an evaluation on the database from our previous trial of 140 patients randomized to either no sedation vs. sedation with a daily interruption of sedatives. A total of 113 patients were included in the previous statistical analysis. Ten patients had pre-existing renal impairments and were excluded. Data were collected from observational cards and blood samples.

**Results:**

A total of 103 patients were included in this retrospective review. We registered an increased urine output in the group receiving no sedation compared to the sedated control group (1.15 ml/kg/hour (0.59 to 1.53) vs. 0.88 ml/kg/hour (0.052 to 1.26), *P *= 0.03). In addition we saw a decrease in the number of patients with renal impairment according to the RIFLE classification (indicating Risk of renal dysfunction; Injury to the kidney; Failure of kidney function, Loss of kidney function and End-stage kidney disease) in the group receiving no sedation compared to the sedated control group (25 (51%) vs. 41 (76%), *P *= 0.012). The difference in the two groups with respect to mean arterial blood pressure, fluid balance and use of vasoactive drugs was not significant.

**Conclusions:**

A no sedation strategy to patients undergoing mechanical ventilation increases the urine output and decreases the number of patients with renal impairments.

**Trial registration:**

ClinicalTrials.gov registration number NCT00466492.

## Introduction

Sedation is used in critically ill patients receiving mechanical ventilation to bring the patients comfort and facilitate mechanical ventilation during intensive care stay [1]. The intention is to sedate and thereby depress the central nervous system (CNS). But sedation not only affects the brain, it also has an effect on many other organs. A common complication to bolus doses of sedative drugs is a decrease in blood pressure. Counter measures are often applied such as infusion of intravenous fluid and initiation of vasopressor drugs to keep the blood pressure within normal range. The effect of continuous use of sedation, compared to a no sedation strategy, on organ function has not yet been described.

Kress and colleagues showed that a daily interruption of sedatives reduced the time patients receive mechanical ventilation and reduced the intensive care length of stay [2]. However, no difference was found with respect to total hospital length of stay. The same group also made a retrospective evaluation of the number of complications between the two study groups: sedation with daily interruption of sedatives or continuous sedation without daily interruption of sedatives [3]. They were able to report a higher number of complications in the continuously sedated control group. Girard and colleagues have shown that combining both a daily interruption of sedatives and a spontaneous breathing trial, compared to only spontaneous breathing, not only reduced time in mechanical ventilation but also decreased total hospital length of stay and 365 days mortality [4].

We have recently published a trial showing that the use of a no sedation strategy to critically ill patients requiring mechanical ventilation increased the number of ventilator free days and decreased both intensive care unit and total hospital length of stay [5]. Especially the observation that a reduction in sedation in the intensive care unit also reduced total hospital length of stay made us perform this not *a priori *defined *post hoc *analysis of data from the original study. We observed that patients from the sedated control group more often developed a degree of renal impairment compared to patients from the awake intervention group. To further elucidate the effect of sedation is vital for our understanding of the consequences with routine use of sedation. Our main hypothesis in this subgroup analysis was that a strategy with no sedation would improve hemodynamic stability (mean arterial pressure); decrease the need for vasoactive drugs; diminish the need for extra fluid; and improve renal function.

## Materials and methods

### Patients and study intervention

In the original study we included patients who were expected to receive mechanical ventilation for more than 24 hours. The inclusion criteria were: age above 17 years, not pregnant, and not in need of sedation because of increased intracranial pressure or undergoing therapeutic hypothermia. We excluded patients who were not expected to wake up with or without sedation (coma hepaticum or neurological coma). A total of 140 patients were randomized in two groups: 70 patients in the awake intervention group and 70 patients in the sedated control group. The intervention group received no sedation except for bolus doses of morphine. The sedated control group also received bolus doses of morphine and infusion of propofol titrated to reach a RAMSAY score of 3 to 4 [6]. After 48 hours propofol was changed to midazolam. In the sedated control group, we performed a daily interruption of sedatives as described by Kress *et al. *[2]. Patients who either had their endotracheal tube successfully removed or died within 48 hours were excluded from the statistical analysis. In this subgroup analysis, patients with a prior history of renal insufficiency (glomerular filtration rate (GFR) below 60 ml/minutes for more than three months [7]) or prior dependency on intermittent dialysis were excluded. This database was provided to investigators (RRJ and JOP) not involved in the original study. They then reviewed all patients' original observation charts. They had no direct knowledge of the randomized treatment but they were not completely blinded because all infusions (including sedatives) could be seen on the observational charts.

The original study was approved by the local ethics committee and informed consent was obtained from each patient or representatives.

### Data collection

Baseline data (age, gender, weight, Acute Physiology and Chronic Health Evaluation (APACHE II) and Sequential Organ Failure Assessment (SOFA) at Day 1) were recorded. The time each patient was dependent on controlled ventilation was also recorded as a baseline value. Once intubated, patients were shifted, as quickly as possible, from pressure control ventilation to pressure support ventilation. This modus is the standard ventilation modus in our department. Mean arterial blood pressure (four times a day at 6 o clock in the morning, noon, 6 o clock in the evening and midnight), use of vasopressor or inotropic drugs, urine output, total amount of fluids was recorded. Results of serum levels of creatinine were also recorded. The RIFLE criteria were adopted from the Acute Kidney Injury Network (AKIN) [8]. The RIFLE classification was defined as follows: RIFLE criteria Risk is defined as: increase in serum creatinine of ≥26.4 μmol/l (150 to 200% of baseline) or urine output of <0.5 ml/kg/h for >6 h. RIFLE criteria Injury is defined as: increase in serum creatinine to >200 to 300% of baseline or urine output <0.5 ml/kg/h for >12 h. RIFLE criteria Failure is defined as: increase in serum creatinine to >300% of baseline (serum creatinine ≥354 μmol/l with an acute rise of at least 44 μmol/l) or urine output <0.3 ml/kg/h for 24 h or anuria for 12 h. Patients who received renal replacement therapy was considered to have met the criteria for failure, irrespective of the stage that they were in at the time of commencement of renal replacement therapy [8].

The highest value according to the RIFLE classification (with respect to urine output and creatinine clearance) was calculated for each patient.

### The study objectives

The primary objective of this study was to test whether a strategy with no sedation would improve hemodynamic stability (mean arterial pressure); decrease the need for vasoactive drugs; diminish the need for extra fluid and lower the risk of acute kidney injury (AKI).

### Statistics

The total number of patients not discharged from the ICU was 44 patients (43%) after a 14-day period. We, therefore, choose to report data from the first 14 days only. The variables: mean arterial blood pressure, fluid balance and urine output are presented in separate figures as mean values calculated for each day in a 14-day period and drawn separately for each group (intervention group with no sedation and control group with sedation). A mean value for each patient up to a period of 14 days was calculated and presented for each variable. Variables for each patient were used for statistical analysis. For each patient a highest RIFLE score was calculated for a 14-day period. The data are presented in a histogram. For statistical testing the patients were divided into two groups: with or without renal impairment.

Data were compared using Wilcoxon Rank-sum test and Chi^2 ^test as appropriate. All tests were performed using: StataCorp. 2007. Stata Statistical Software: Release 10.1. College Station, TX, USA: StataCorp LP.

## Results

**Figure 1:**
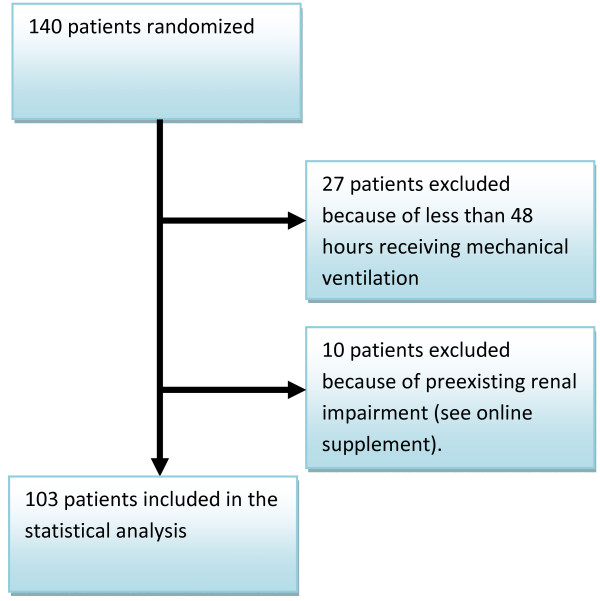
Consort diagram.

We randomized 140 patients from April 2007 to December 2008. Twenty-seven patients were discontinued from mechanical ventilation within 48 hours either because of death or successful extubation. Ten patients had an existing renal insufficiency and were excluded from this subgroup analysis. A total of 103 patients were included in the analysis (Figure 1). Baseline data are shown in Table 1. Patients are comparable with respect to age, weight, APACHE II, SOFA score, S-creatinine and urine output at Day 1. We found no difference in the cumulated time patients were dependent on controlled ventilation modus compared to support modus. "Patients at risk" and still admitted in the intensive care unit are shown in Table 2. In a 14-day period we found no statistically significant difference in the mean arterial blood pressure between the two groups (Table 3).

**Table 1:** Baseline characteristics on admission to intensive care unit

	Intervention group (*n *= 49)	Control group (*n *= 54)	*P-*value
**Age (years)**	67 (55 to 73)	64.5 (56 to 74)	**0.88**
**Gender (female)**	13 (26.5%)	23 (42.6%)	**0.08**
**Weight (kg)**	80 (70 to 92)	76 (69 to 91)	**0.40**
**APACHE II score**	24 (19 to 30)	26 (22 to 29)	**0.30**
**SOFA score (Day 1)**	7 (5 to 11)	9 (6 to 12)	**0.34**
**Urine output ml/kg/hour Day 1**	0.35 (0.13 to 0.82)	0.25 (0.11 to 0.85)	**0.53**
**Serum creatinine mikromol/l (Day 1)**	122 (93 to 181)	157 (99 to 219.5)	**0.26**

Data are in median (IQR) or number (%). APACHE II: Acute Physiology and Chronic Health Evaluation; SOFA: Sequential Organ Failure Assessment.

**Table 2:** Number of patients at risk (admitted to ICU) in a 14-day period

Day	1	2	3	4	5	6	7	8	9	10	11	12	13	14
**Not sedated**	49	49	46	43	37	32	29	26	25	24	22	21	20	18
**Sedated**	54	54	49	46	45	44	42	40	38	36	33	31	27	26

**Table 3:** Results table

	Intervention group (*n *= 49)	Control group (*n *= 54)	*P *value
Arterial blood pressure mmHg	81 (75 to 88)	77 (75 to 85)	0.24
Noradrenaline ml/hour/day*	0.11 (0 to 0.68)	0.07 (0 to 0.74)	0.64
Morphine mikrog/kg/hour $	5.16 (1.25 to 11.07)	4.51 (2.02 to 6.40)	0.40
Diuretics (furosemide) mg/kg/day	0.23 (0.09 to 0.38)	0.19 (0.05 to 0.38)	0.38
Fluid balance ml/kg/day	4.58 ((-0.13) to 9.88)	9.70 ((-2.03) to 22.79)	0.13
Total cumulative fluid balance ml/kg	24.46 (0 to 50.42)	61.38 (0 to 105.75)	0.38
Urine output ml/kg/hour	1.15 (0.59 to 1.53)	0.88 (0.052 to 1.26)	0.03
Number of patients starting CRRT	18 (33%)	21 (43%)	0.32
Number of patients with renal impairment**	25 (51%)	41 (76%)	0.012
Cumulated time dependent on controlled ventilation Days #	0.46 (0.13 to 1.79)	0.46 (0.21 to 2.25)	0.56

All values in median (IQR) or number (%). Each value is calculated from a mean for each patient in the first 14 days of their intensive care stay. *One milliliter corresponds to 0.01 μg/kg/minute. CRRT: continuous renal replacement therapy. $, Morphine median value for entire period of mechanical ventilation. ** Renal impairment according to RIFLE class Risk, Injury or Failure. #, Time before patients was shifted to support ventilation.

The use of vasopressor is shown in Table 3. Noradrenaline was the most used vasoactive drug in these patients. The use of dopamine, dobutamine and adrenaline was very low in both groups (data not shown). No difference was found in the use of vasopressors. No difference between the groups was found in the use of morphine or diuretics (Table 3).

**Figure 2:**
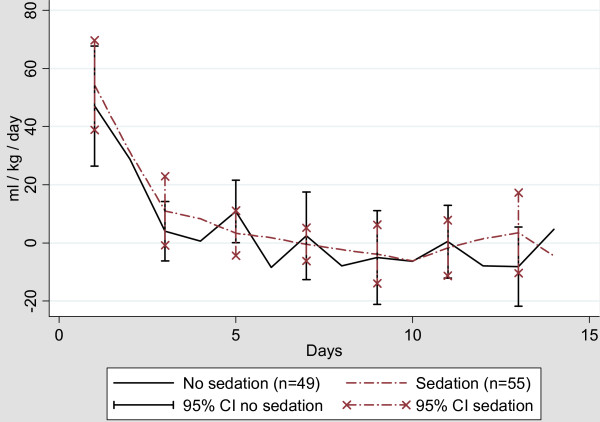
**Daily fluid balance in ml/kg/day**.

Both groups of patients were in a positive fluid balance the first five days (Figure 2). After Day 5, both groups were in zero to slight negative fluid balance. Although it did not reach statistical significance, a median value of 9.70 ml/kg/day was found in the sedated control group compared to 4.58 ml/kg/day in the awake intervention group (*P *= 0.13). In addition, the cumulative fluid balance was higher in the sedated group 61.38 (0 to 105.75) ml/kg) compared to 24.46 (0 to 50.42) ml/kg in the awake intervention group. This difference, however, did not reach statistical significance.

**Figure 3:**
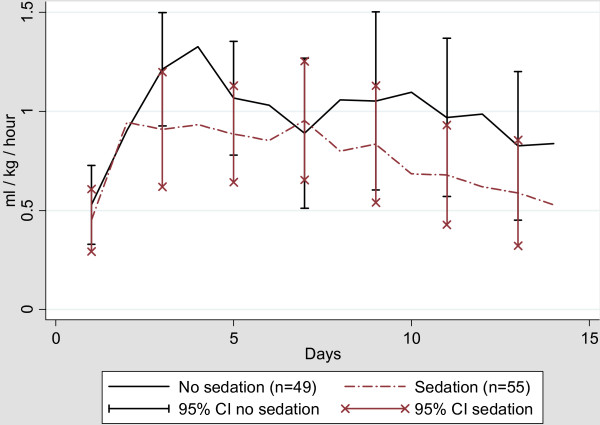
**Daily mean urine output in ml/kg/hour**.

We found a significantly higher urine output in the awake group during the first 14 days compared to the sedated control group (*P *= 0.03) (Figure 3 and Table 3). This was seen even though we found no difference in the use of colloids (data not shown) or diuretics between the two groups.

**Figure 4:**
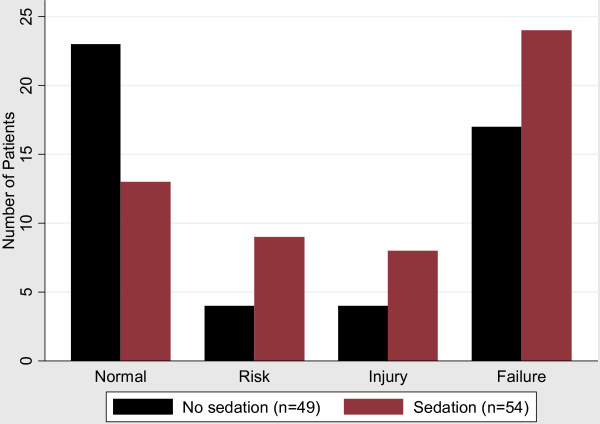
**Patients divided in RIFLE classes, highest value in a 14-day period**.

The renal function expressed according to the RIFLE classification is shown in Figure 4. The highest value for each patient in a 14-day period is shown in the histogram. In the first group of patients with normal renal function there are a higher number of patients from the non-sedated intervention group. In the other three groups with different degrees of acute kidney injury (Risk, Injury or Failure) there is a higher number of patients from the sedated group. This difference is statistically significant (*P *= 0.012). The part of the RIFLE classification with the highest impact was the low urine output. Only one patient reached a higher RIFLE class solely because of a rise in serum creatinine.

There was no difference observed in the number of patients treated with continuous renal replacement therapy. Eighteen patients from the awake intervention group started continuous renal replacement therapy and 21 in the sedated control group (*P *= 0.32) (Table 3).

## Discussion

We found that the use of sedation decreased the urine output and increased the frequency of acute kidney injuries expressed according to the RIFLE classification. This finding is interesting because Bagshaw and colleagues observed an association between increase in hospital mortality and successive increase in severity of RIFLE category [9]. The increase in acute kidney injuries in the sedated control group is, therefore, suggestive of a poorer outcome and might explain a part of the prolonged hospital length of stay in this group. A significant increase in the number of the more severe cases of patients with acute kidney injuries who needed continuous renal replacement therapy was not demonstrated. However, the RIFLE classification is a very sensitive parameter and not all patients classified as having AKI according to the RIFLE classification receives renal replacement therapy during their ICU stay.

No difference was found in blood pressure between the two groups. One might *a priori *suspect that this lack of difference in blood pressure (data not shown) could be explained by an increased use of vasoactive drugs and fluids in the sedated control group. Surprisingly we found no statistical difference in the use of vasoactive drugs, fluid balance, and the use of colloids or diuretics during the first 14 days.

The mechanism behind the renal impairment observed in our study is not known. A decrease in the microcirculation within the kidneys following sedation might be part of the explanation. Sedation probably has an impact on other organs such as the gastrointestinal tract, kidneys and lungs. It is difficult to monitor the end organ perfusion and a clear explanation cannot be concluded with our present data. It is, however, likely that our present findings with decreased urine output and increased risk of AKI evaluated in with the RIFLE classification in the sedated group have a multifactorial origin from the use of sedation. Koch and colleagues found that the use of propofol in patients undergoing elective surgery reduced the microcirculation [10]. Although we only used propofol for a maximum of 48 hours and then switched to midazolom, a reduction in microcirculation could explain some of our findings. The observed renal impairment induced by the use of sedative drugs in the present study might explain part of the prolonged hospital length of stay.

The use of sedation has several other disadvantages. Sedation eliminates the possibility to clinically observe the cerebral function of patients and complicates the ability to detect delirium since Richmond Agitation-Sedation Scale (RASS) needs to be at least -3 or above to use the CAM-ICU score to detect delirium [11-13]. CNS function is a very important parameter in the continuous observation of the critically ill patient. The drugs used to induce sedation are not organ specific. The use of sedation could cause a need for vasopressors or fluids to counteract the vasodilatation introduced by the use of sedation in the critically ill patient. The use of vasopressor agents to counteract a reduction in blood pressure does not increase the microcirculation in critically ill patients. This was demonstrated by Dubin and colleagues who increased the mean arterial pressure above 65 mmHg in patients with septic shock [14]. This elevation of blood pressure did not increase the microcirculation.

The use of pressure support ventilation as the preferred ventilator strategy differs from the generally used ventilation modus for critically ill patients, which is controlled ventilation [15]. It is our policy to shift the ventilator modus to pressure support as soon as the patients are able to trigger the ventilator. *A priori *one would expect the use of sedation to decrease the patient's ability to interact with the ventilator, thereby prolonging the need for mechanical ventilation for the sedated patients. This would also be an obvious factor in explaining the observed renal effects of sedation. Surprisingly, we found no difference between the two groups in the time patients needed controlled ventilation. As earlier reported, the total amount of sedatives used in the sedated group was low which could explain the ability to trigger the ventilator [5]. However, as earlier demonstrated, the total time receiving mechanical ventilation (controlled ventilation and pressure support) was increased by the use of sedation.

This study holds several limitations. It was not an *a priori *planned study and only included a single center with relatively few patients. It was, therefore, not powered to detect a difference in renal function or fluid balance. The observed difference in urine output could be because of a type I error and simply an observation done by chance. Also, the fact that no differences were found in other parameters such as blood pressure, fluid balance and use of vasopressors is challenging. However, the study was conducted in an institution with no sedation as standard care. The sedated control group was less sedated than centers with routine use of sedation. Still we found a difference in urine output which is an important message with the widespread routine use sedatives [16,17].

We choose only to report data from up to a 14-day period because of the very different number of days patients were admitted to the intensive care department (see Table 2). Reporting and analysing a longer time interval would hold a risk that data from only a few patients would be amplified and perhaps give a misleading result. The SAFE (Saline versus Albumin Fluid Evaluation) Study Investigators recently published a subgroup analysis only reporting data from the first seven days [18]. In our opinion, the 14 days represents a good compromise between an acceptable time frame and a representative number of patients (Table 2).

Prospective randomized studies are difficult and time consuming to conduct but they are still the gold standard for proving or rejecting new knowledge. A not *a priori *defined finding from a prospective single center study always holds a risk of being a random finding (type 1 error). However, subgroup analysis data from prospective randomized trials are important in designing new studies aimed at proving or rejecting a hypothesis. A prospective randomized multicenter study, powered to detect difference in fluid balance, renal function and mortality is now warranted.

## Conclusions

In the present study we showed that a strategy with no sedation resulted in less renal impairment evaluated by urine output and RIFLE classification compared to the use of sedation. No difference in blood pressure or the need for vasopressor agents was observed. A large prospective multicenter study with renal impairment as one of the primary defined endpoints to confirm whether sedation causes renal impairment is now warranted.

## Key messages

• Routine use of sedatives prolonged the time patients received mechanical ventilation and length of stay

• This *post hoc *analysis suggests an increase in acute kidney injury with the use of sedation compared to a strategy with no sedation

• No significant difference was found with respect to the use of vasopressors or fluid balance

• A larger multicenter study is needed to verify the thesis that sedation increase the risk of acute kidney injury

## Abbreviations

AKIN: Acute Kidney Injury Network; APACHE II: Acute Physiology and Chronic Health Evaluation; CAM-ICU: confusion assessment method for the intensive care unit; GFR: glomerular filtration rate; RASS: Richmond Agitation-Sedation Scale; RIFLE: acronym indicating Risk of renal dysfunction, Injury to the kidney, Failure of kidney function, Loss of kidney function and End-stage kidney disease criteria; SOFA: Sequential Organ Failure Assessment.

## Competing interests

The authors declare that they have no competing interests.

## Authors' contributions

TS and PT conceived and designed the study and drafted the manuscript. RRJ and JOP collected data. All authors contributed to the review and revisions of the manuscript. All authors read and approved the final manuscript.
